# Supplementary material for: Emergent effects of synaptic connectivity on the dynamics of global and local slow waves in a large-scale thalamocortical network model of the human brain
Source: PLoS Comput Biol. 2024 Jul 19;20(7):e1012245. doi: 10.1371/journal.pcbi.1012245 (PMC11290683; doi:10.1371/journal.pcbi.1012245)
Supplement: S1 Text — Fig A. Neuronal activity in all 6 layers during the Global SO model from Fig 2. Voltage traces in mV for two different cells are shown on the left panels, with individual spikes marked under each trace in the same color. Fig B. Activity of pyramidal, inhibitory and thalamic RE and TC neurons in baseline model in Fig 2B. Voltage traces in mV for two different cells are shown on the left panels, with individual spikes marked under each trace in the same color. Fig C. Activity of cortical cells in all layers in mixed model. Voltage traces in mV for two different cells are shown on the left panels, with individual spikes marked under each trace in the same color. Fig D. Activity of pyramidal, inhibitory, and thalamic cells in the mixed model. Voltage traces in mV for two different cells are shown on the left panels, with individual spikes marked under each trace in the same color. Fig E. Activity of pyramidal and inhibitory neurons in the Activity by Layer and Cell Type: Mixed local/global model in Fig D, with the thalamus isolated from the cortex. Voltage traces in mV for two different cells are shown on the left panels, with individual spikes marked under each trace in the same color. Fig F. Local activity for P = 0.1, with 90% connections removed. A) Ten cortical areas with a 5mm radius, that were used to calculate local dynamics. B) Average membrane voltage of layer II neurons, as in Fig 3e.2. C-E) For each region in (A), subpanels show: (C) the single-cell voltage for two neurons in the area, (D) the local field potential (LFP) for the 5mm area, and (E) heatmap of individual voltages of all neurons in the area. Up states are largely synchronized across all 5 regions. F) Latency map for each Up state in the P = 0.1 simulation. Even with very sparse connectivity, Up states spread to the whole cortex. Participation was reduced uniformly to about 70% compare to nearly 100% participation when all connections are present (compare to Fig 2D). Fig G. Effect of Synaptic Del [file pcbi.1012245.s001.pdf]

## S1 Text

### Activity by Layer and Cell Type: Global SO model

Fig A shows the activity of all 6 modeled layers baseline SO simulation in Fig 2. Activity is largely synchronized across layer. Layer 4 is consistently less active than other layers, which is likely a result of L4 PY cells having within-column connections from exclusively one other layer (L6, see Fig 1E)

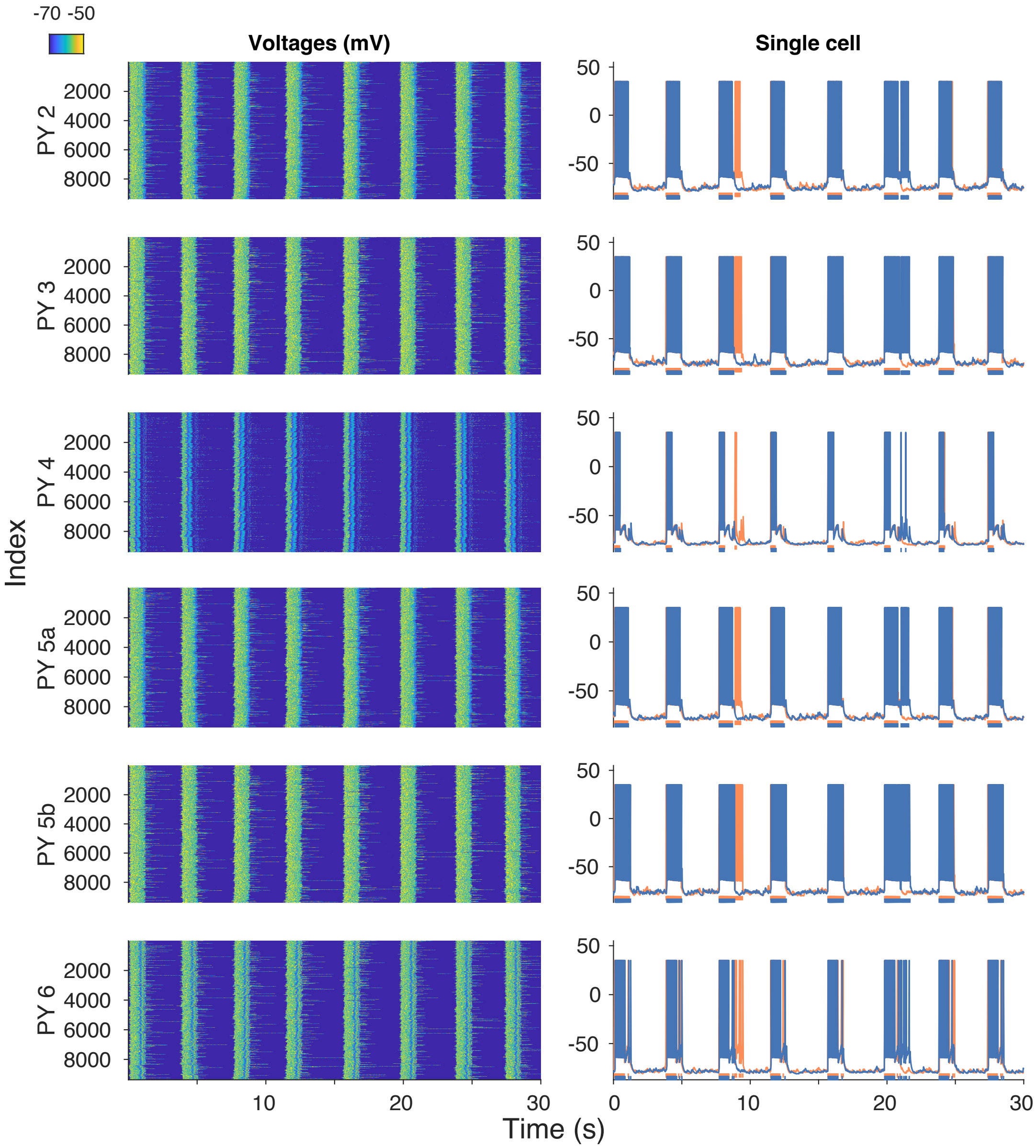


**Fig A.** Neuronal activity in all 6 layers during the Global SO model from Fig 2. Voltage traces in mV for two different cells are shown on the left panels, with individual spikes marked under each trace in the same color.

Fig B shows the activity of modeled core and matrix thalamocortical (TC, TCa) and reticular (RE, REa) cells as well as inhibitory (IN) cells.

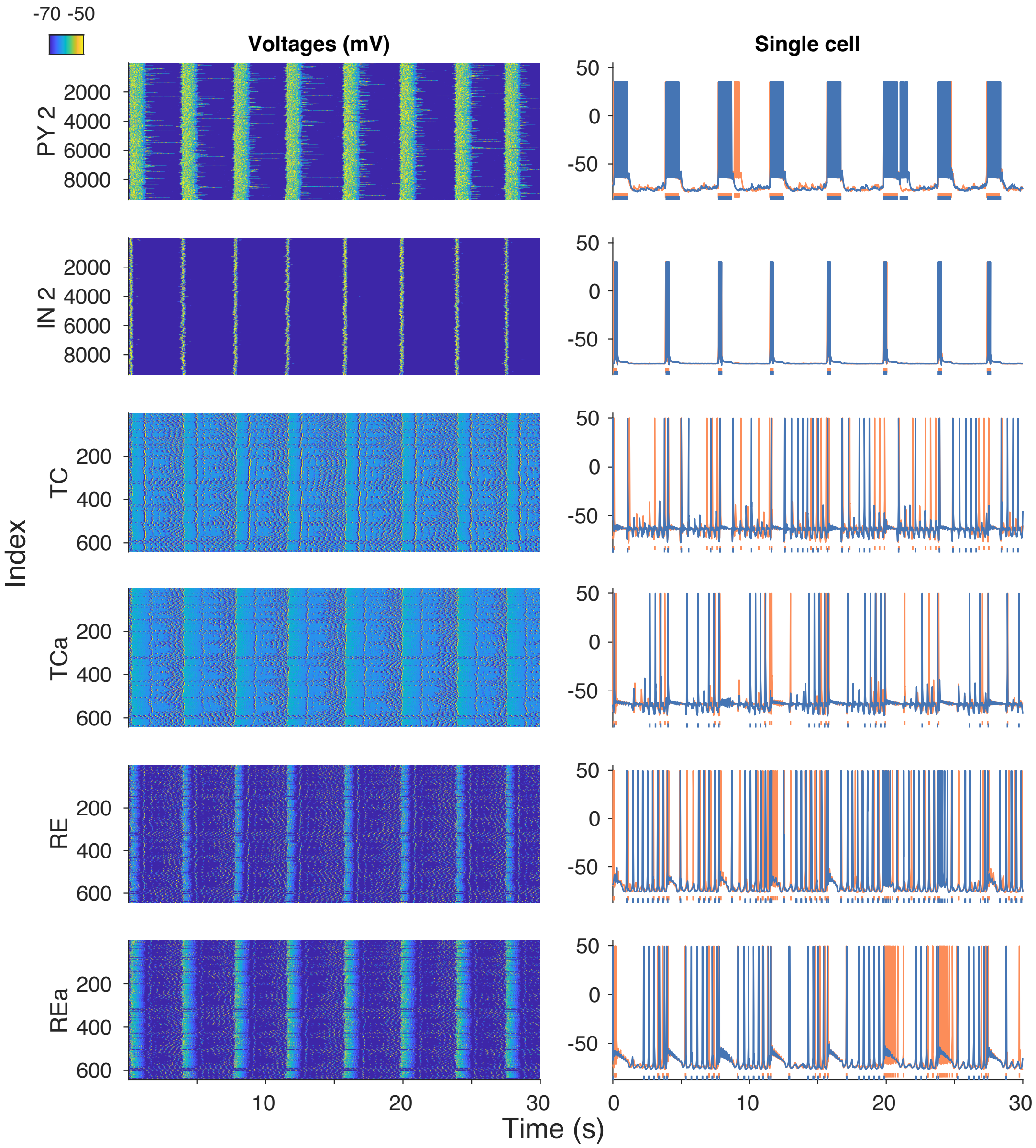


**Fig B.** Activity of pyramidal, inhibitory and thalamic RE and TC neurons in baseline model in Fig 2B.Voltage traces in mV for two different cells are shown on the left panels, with individual spikes marked under each trace in the same color.

## Activity by Layer and Cell Type: Mixed local/global model

Fig C shows all layers of cortical cells in the mixed model (Local vs Global Slow-Waves) with global and local Up states. Similar to the Activity by Layer and Cell Type: Global SO model, layers are largely synchronized, with a relatively less active L4.

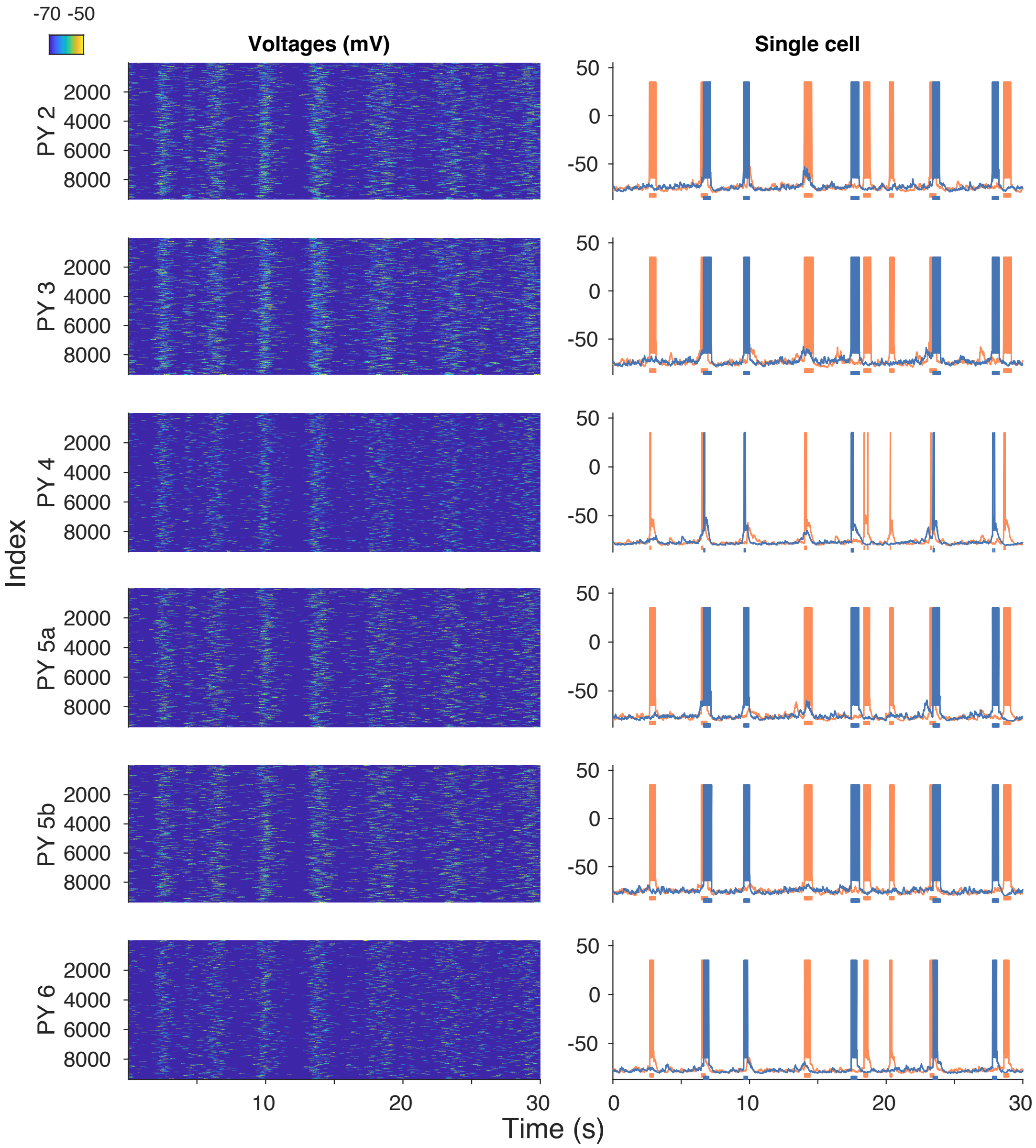


**Fig C.** Activity of cortical cells in all layers in mixed model.Voltage traces in mV for two different cells are shown on the left panels, with individual spikes marked under each trace in the same color.

Fig D additionally shows the activity of inhibitory and thalamic core and matrix cells (TC, TCa, RE and REa).

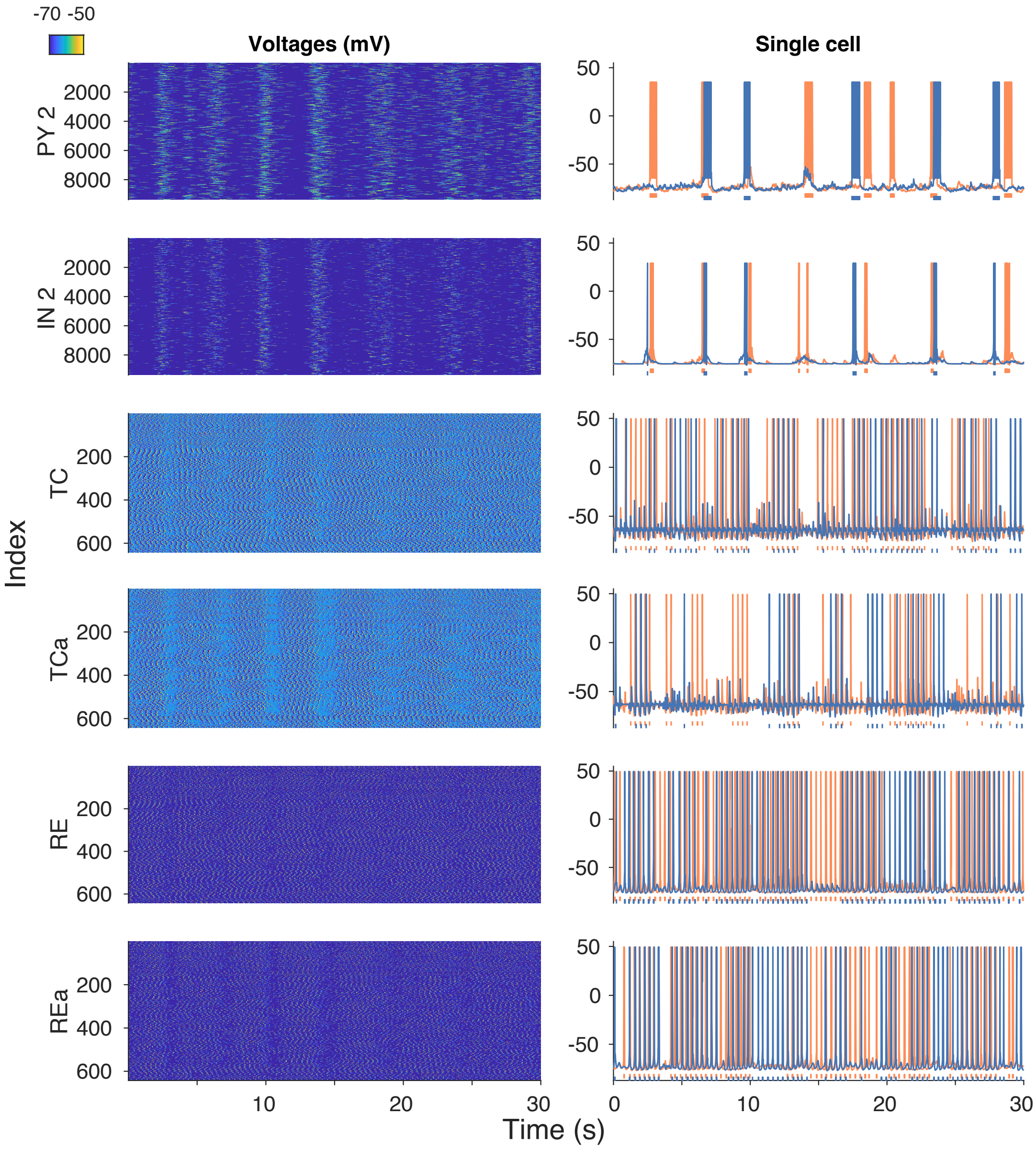


**Fig D.** Activity of pyramidal, inhibitory, and thalamic cells in the mixed model. Voltage traces in mV for two different cells are shown on the left panels, with individual spikes marked under each trace in the same color.

## Effect of thalamus on SO synchronization

Fig E shows activity of pyramidal cell and inhibitory INs in the Activity by Layer and Cell Type: Mixed local/global model when the thalamus and cortex are isolated. SO activity is maintained in cortical cells, but less synchronized than in simulations including the thalamus (compare to Fig D). Thalamic cells (not shown) remain inactive in the absence of cortical inputs. On the other hand, removing the thalamus in the Activity by Layer and Cell Type: Global SO model does not produce an appreciable effect, likely because the impact of thalamic inputs diminishes relative to the elevated cortical synaptic weights, which are high enough to produce synchronous Up states.

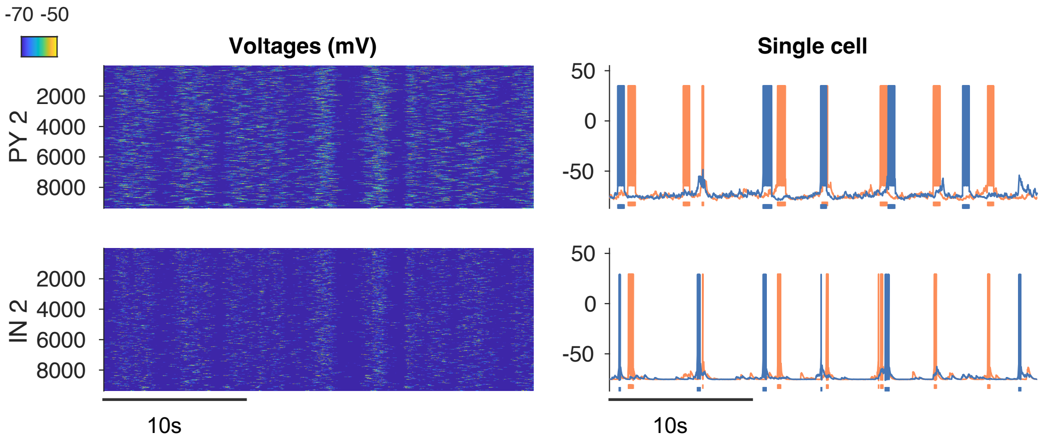


**Fig E.** Activity of pyramidal and inhibitory neurons in the Activity by Layer and Cell Type: Mixed local/global model in Fig D, with the thalamus isolated from the cortex. Voltage traces in mV for two different cells are shown on the left panels, with individual spikes marked under each trace in the same color.

## Effects of connections density, range and delays

Fig F shows further details into the slow-wave dynamics in the case of extreme density loss ($P = 0.1$), zooming into ten cortical regions with a 5mm radius each (Fig FA). Single cells and LFPs in different regions throughout the cortex (Fig FC-D) showed synchronized Up states, although many neurons were not active during individual Up states (Fig FC, E). The local heterogeneity in activation patterns induced by the extreme sparsity of connections is evident in Fig FE, which shows many silent neurons in the immediate vicinity of active cells. This generated the extremely granular participation seen in the latency maps (Fig FF). Even though each Up state encompassed cells in the whole cortex (high spread), a large fraction of cells distributed through the entire cortex did not participate in slow-waves (decreased participation). This model can further be seen in S2 Video. Initiation was diffused with respect to the baseline simulation, to the point where a single meaningful estimate of propagation speed for each Up state could no longer be computed.

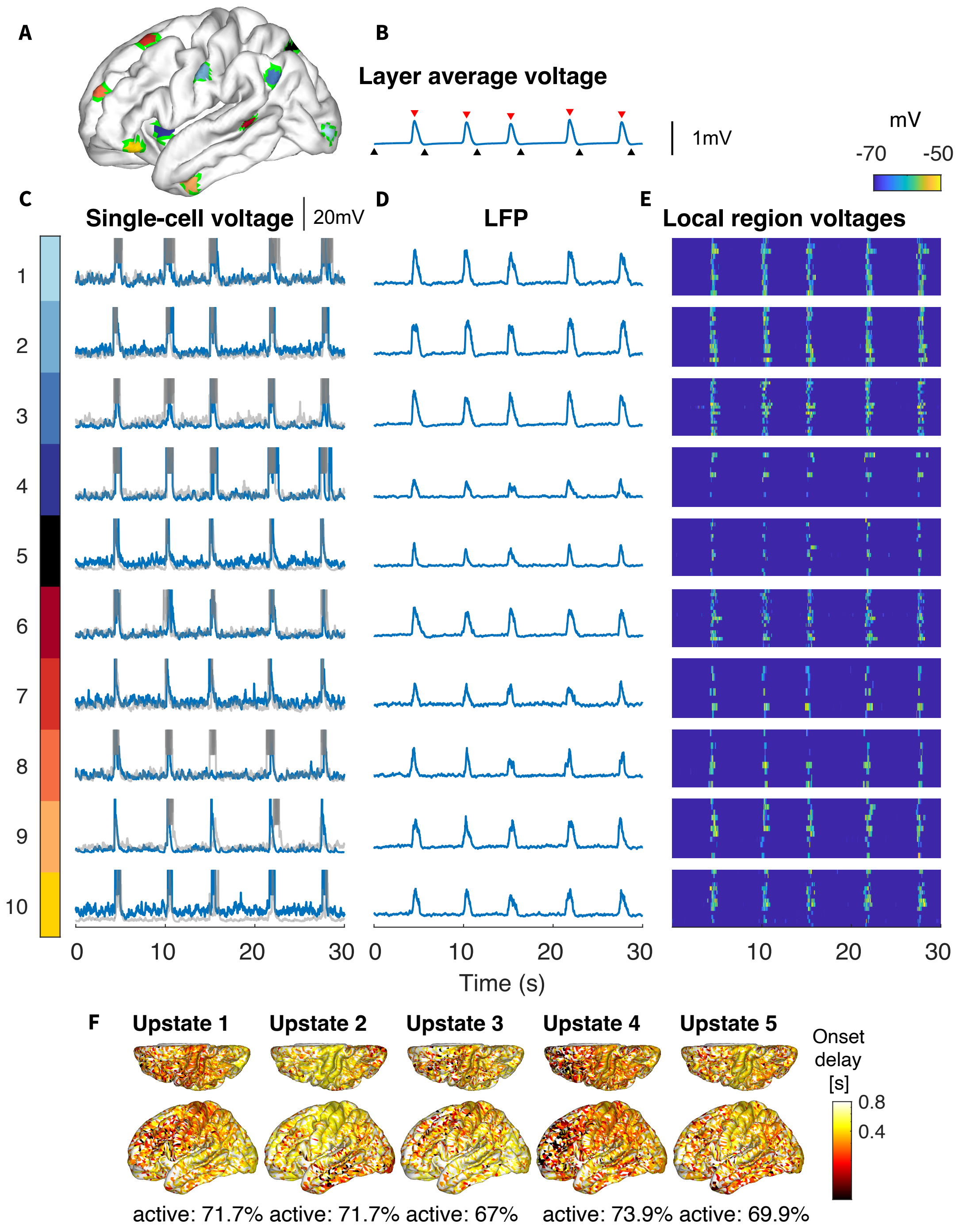


**Fig F.** Local activity for $P = 0.1$, with 90% connections removed. A) Ten cortical areas with a 5mm radius, that were used to calculate local dynamics. B) Average membrane voltage of layer II neurons, as in Fig 3e.2. C-E) For each region in (A), subpanels show: (C) the single-cell voltage for two neurons in the area , (D) the local field potential (LFP) for the 5mm area, and (E) heatmap of individual voltages of all neurons in the area. Up states are largely synchronized across all 5 regions. F) Latency map for each Up state in the $P = 0.1$ simulation. Even with very sparse connectivity, Up states spread to the whole cortex. Participation was reduced uniformly to about 70% compare to nearly 100% participation when all connections are present (compare to Fig 2D)

Fig G shows the effect of modifying synaptic delays on network behavior. Delays were changed in two ways: (1) by setting a maximum delay and scaling all other delays with distance and (2) by imposing a fixed delay value for all connections and varying this value (see Connection Delay for details)

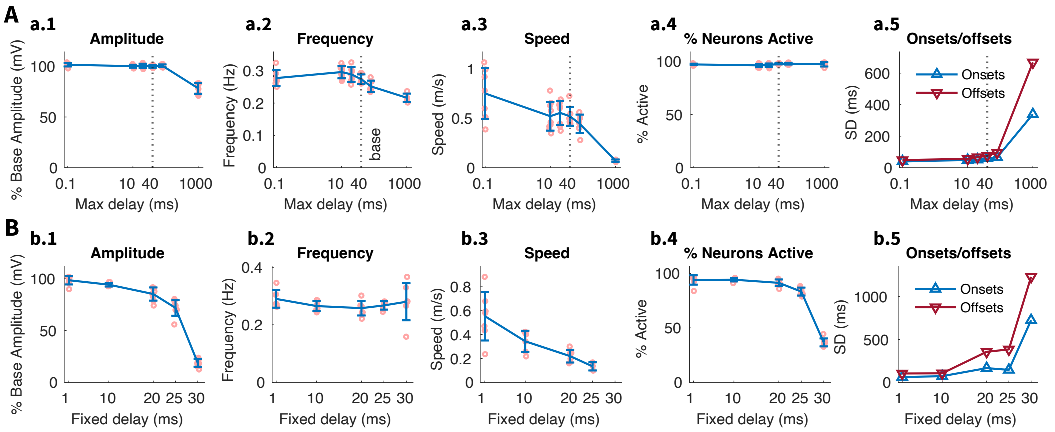


**Fig G.** Effect of Synaptic Delay, either via setting a uniform delay (A) or scaling the max delay (B) on SO dynamics. (A and B) Summary plots of frequency, amplitude, and onset / offset spread. Decreasing the max scaled delay to 0.1 ms (from 40 ms) has no effect, while increasing the max scaled delay up to 1 second shows only minor changes in frequency frequency and amplitude but notably increased onset/offset synchrony. Low uniform delays similarly showed no effect, with loss of amplitude and synchrony only seen at 30 ms (most delays were previously under 2 ms).

## Graph Properties

Fig H and Fig I show activity maps and graph properties of the networks for the Global slow wave simulation in Fig 2 and for the restricted range $R = 2.5mm$ simulation in Fig 5. Activity characteristics (percent time active, mean onset/offset delays) are shown for pyramidal neurons in cortical layer 2 only for 30 seconds of simulation time, as in Slow Wave Characterization and Connection Range. The percent time active for each neuron is defined as the fraction of the simulation time that the neuron spends in an Up state (above $-65$mV, see Latency and participation). The mean onset/offset delay for each cell is an average of its onset/offset values across Up states (see Latency and participation for details on onsets/offsets).

The graph properties (strongly/weakly connected components, in/out degree, and centrality) are based on structural connectivity only, independent of activity during simulations. A weighted directed graph is constructed for each network, where each column is a node and the edge from node $i$ to node $j$ has an associated weight equal to the number of synaptic connections from column $i$ to column $j$. For each graph, Fig H and Fig I show: the number of connected components (two nodes belong to the same weak or strong component only if there is a path connecting them in either or both directions, respectively); the normalized in/out degree for each node (number of edges with that node as the target/source); and the normalized in/out-degree centrality (number of connections to/from the column, equal to the total weight of incoming/outgoing edges).

For the full-connectivity network, initiation zones (characterized by low mean onset delay, Fig H *second row*) generally correspond to lower in-degree and out-degree regions (Fig H *bottom row*) . For the local connectivity network, regions with high percent time active (corresponding to participating regions during Up states in Fig 5F and Fig I, *second row*) have high in/out-degree and centrality (Fig I, *bottom rows*) .

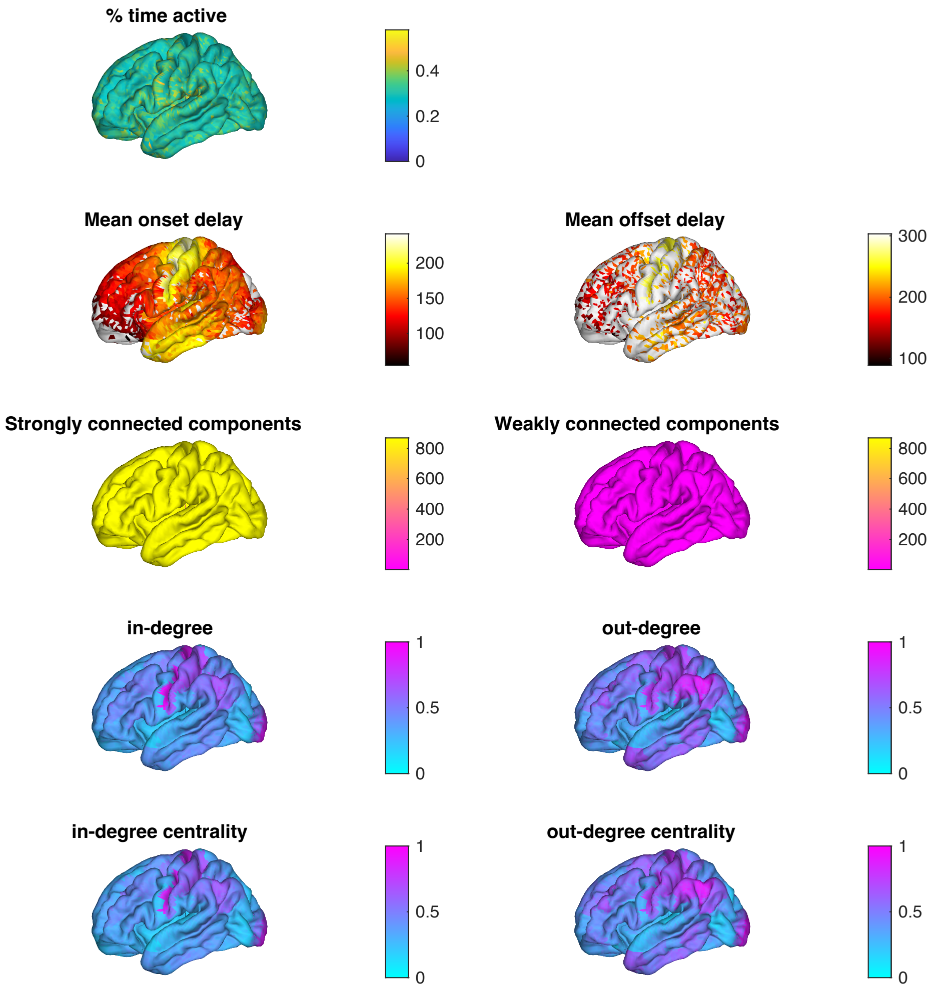


**Fig H.** Activity maps and graph properties for the full-connectivity, Global SO network in Fig 2. Rows from top to bottom show (1) the percent of simulation time that each pyramidal cell in layer 2 spends in an Up state; (2) the mean onset/offset delay across Up states for each pyramidal cell in layer 2; (3) the number of strongly/weakly connected components in the structural connectivity graph, constructed with each cortical column as a node and each directed edge weight as the number of synaptic connections from one column to another; (4) the normalized in/out-degree for each node in the graph; and (5) the normalized in/out-degree centrality for each node in the graph, defined as the number of synaptic connections to/from that column (see Graph Properties for details). Initiation zones (low mean onset delay) at the front and back and have generally lower in-degree and out-degree than areas in the middle.

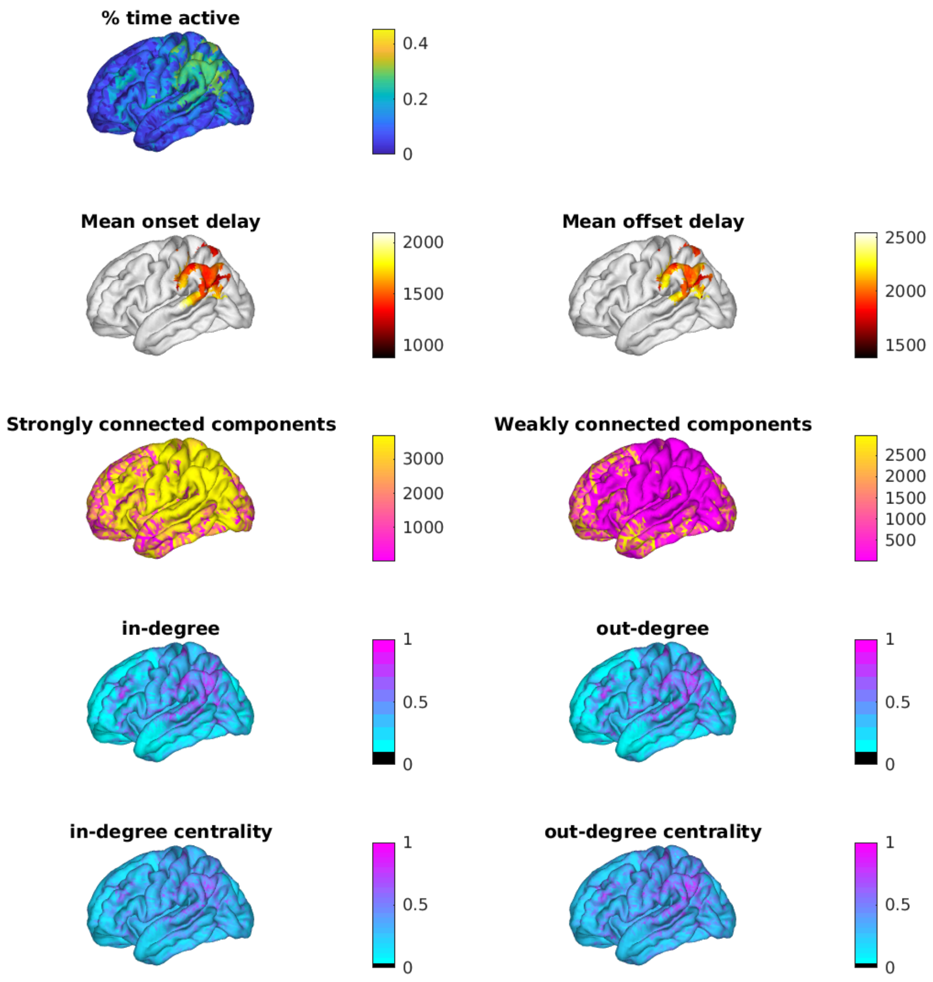


**Fig I.** Activity maps and graph properties for the $R = 2.5$mm network in Fig 5. Rows from top to bottom show (1) the percent of simulation time that each pyramidal cell in layer 2 spends in an Up state; (2) the mean onset/offset delay across Up states for each pyramidal cell in layer 2; (3) the number of strongly/weakly connected components in the structural connectivity graph, constructed with each cortical column as a node and each directed edge weight as the number of synaptic connections from one column to another; (4) the normalized in/out-degree for each node in the graph; and (5) the normalized in/out-degree centrality for each node in the graph, defined as the number of synaptic connections to/from that column (see Graph Properties for details). Regions with high percent time active have high in/out-degree and centrality

## Model Coherence

Table A lists all frequencies used for coherence analysis.

| Frequency (Hz) | Bandwidth (Hz) |
|---|---|
| **0.5** | 0.5 |
| **1** | 0.5 |
| **1.5** | 0.5 |
| **2** | 0.5 |
| **2.5** | 0.5 |
| **3** | 0.5 |
| **3.5** | 0.5 |
| **4** | 0.5 |
| **4.5** | 0.5 |
| **5** | 0.5 |
| **6** | 1 |
| **7** | 1 |
| **8** | 1 |
| **9** | 1 |
| **10** | 1 |
| **12** | 2 |
| **14** | 2 |
| **16** | 2 |
| **18** | 2 |
| **20** | 2 |
| **22** | 2 |
| **24** | 2 |
| **26** | 2 |
| **30** | 2 |
| **35** | 5 |
| **40** | 5 |
| **50** | 10 |
| **60** | 10 |
| **70** | 10 |
| **80** | 10 |
| **90** | 10 |
| **100** | 10 |

**Table A.** Peaks and 3 dB attenuation bandwidths for the second-order resonator filters used to extract narrow-band signals.

**Video S1.** The base global slow wave sleep model over 30 seconds of simulated time. The top plot shows Layer 2 cell voltages across the cortex, while the bottom plot shows the average voltage trace.

**Video S2.** The slow wave sleep model with a connection density of P = 0.1 over 30 seconds of simulated time. The top plot shows Layer 2 cell voltages across the cortex, while the bottom plot shows the average voltage trace.

**Video S3.** The slow wave sleep model with a connection range of R = 2.5 mm over 20 seconds of simulated time. The top plot shows Layer 2 cell voltages across the cortex, while the bottom plot shows the average voltage trace.

**Video S4.** The slow wave sleep model with connections longer than 10mm reduced 5-fold over 105

seconds of simulated time. The top plot shows Layer 2 cell voltages across the cortex, while the bottom plot shows the average voltage trace.
